# Thinking Outside the Box: The Interventional Surgeon

**DOI:** 10.21470/1678-9741-2023-0207

**Published:** 2024-03-20

**Authors:** Adnaldo da Silveira Maia, Karlos Jennysson Sousa Soares, Pedro Esteban Ulloa Alvarado, Francisco Victor Alves da Silva, Dayara Hoffmann Mayer, Mauro Henrique Batista Camacho, José Honório de Almeida Palma da Fonseca

**Affiliations:** 1 Department of Cardiovascular Surgery, Instituto Dante Pazzanese de Cardiologia (IDPC), São Paulo, São Paulo, Brazil; 2 Department of Cardiovascular Surgery, Hospital do Coração (HCor), São Paulo, São Paulo, Brazil; 3 Department of Cardiovascular Surgery, Instituto do Coração (InCor), Hospital das Clínicas, Faculdade de Medicina, Universidade de São Paulo, São Paulo, São Paulo, Brazil

**Keywords:** Internship and Residency, Brazil, Surgeons, Heart Diseases, Catheters

## Abstract

Advances in treatment of structural heart disease have been disruptive to cardiovascular surgery, and there have been discussions about how to incorporate these technologies into the surgeons’ therapeutic arsenal. Transcatheter procedures, complex redo interventions, and endovascular aortic approaches are already practiced by cardiovascular surgeons in Brazil. The expansion of these techniques, coupled with recent changes in the country’s medical residency program in cardiovascular surgery, has led to an urgent need to acquire catheter-based skills. In this article, we discuss these aspects in the light of the reality of cardiovascular surgery training in Brazil.

## INTRODUCTION

**Table:** 

Abbreviations, Acronyms & Symbols	
**BJCVS**	**= Brazilian Journal of Cardiovascular Surgery**
**PCI**	**= Percutaneous coronary intervention**
**PGY**	**= Postgraduate year**
**SAVR**	**= Surgical aortic valve replacement**
**SBCCV**	**= Sociedade Brasileira de Cirurgia Cardiovascular**
**TAVI**	**= Transcatheter aortic valve implantation**
**TAVR**	**= Transcatheter aortic valve replacement**

Cardiovascular surgery has undergone numerous changes in the past few years. The beginning of the use of stents for treatment of coronary artery disease and, more recently, the transcatheter approach in the management of structural heart diseases can be considered landmarks for the specialty. In 2022, the scientific community remembered the 20^th^ anniversary of the first transcatheter aortic valve implantation (TAVI) performed by Alain Cribier, this procedure inaugurated a new field of action with several converging areas and a unique opportunity for the therapeutic arsenal of surgeons^[1]^.

However, after decades, some questions remain, especially in the national context. What is the role of the Brazilian cardiovascular surgeon in this “minimally invasive” era? What measures were and are still necessary in the training of future surgeons? Are we at the forefront of endovascular aortic and structural heart disease treatments? Hence, this article seeks to address such issues in the face of this extraordinary evolution, the emergence of new devices and research in this field as well as the understanding that the survival of a specialty permeates the concern about the continuous training of the already established surgeons and the new generations to come.

## A NEW PARADIGM

The traditional cardiac surgeon’s need for adaptation has become increasingly imperious. Nguyen et al.^[2]^ point out that all training must be reviewed in order to allow contact with new transcatheter technologies. The very recent history of vascular surgery can contribute to understand the concept of adaptation. With the development of interventional radiology techniques, patients with peripheral arterial disease could be treated in a less invasive way. In this context, vascular surgeons adopted such technologies in their daily practice as well as changes in training programs and investment in innovation, where such measures revolutionized the specialty as it is practiced today^[2]^.

**Fig. 1:**
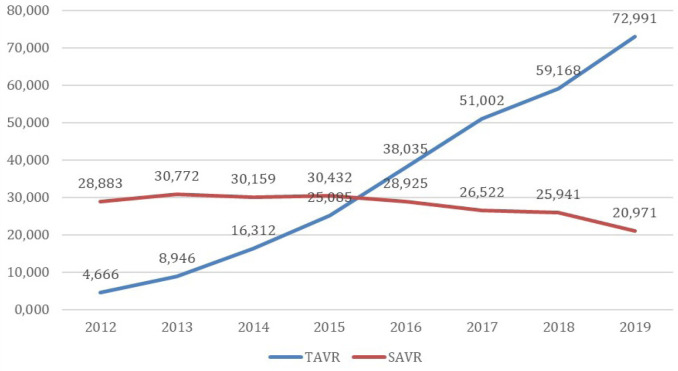
The volume of transcatheter aortic valve replacement (TAVR) exceeded isolated surgical aortic valve replacement (SAVR) between 2015 and 2016, with a decline in isolated SAVR since 2016. Adapted from Carrol et al.^[5]^.

We can say that cardiovascular surgery currently lives in a context of disruptive technologies. This term refers to a technological innovation that produces a change in pre-established models, techniques, and concepts. The development of stents for the aorta and coronary arteries, TAVI, and recent alternatives for mitral and tricuspid valves, as well as valve-in-valve implants, are new approach options and establish a new paradigm for surgeons^[3]^. To understand this fact, let’s face the data. The average number of cardiac surgeries performed in Brazil is approximately 100,000 per year, most of which consist of myocardial revascularization, followed by valve surgery^[4]^. When we evaluate data from the United States of America, the amount of TAVI surpassed the amount of isolated valve replacements in 2015, with a clear rise, even doubling the number of cases in 2019. Two other points are worth mentioning: the expansion of indications and accesses. The Society of Thoracic Surgeons’ record demonstrates a clear expansion in intermediate and low-risk cases, and this fact is associated with the prevalence of transfemoral access in 95.3% of patients^[5]^ (Figure 1).

**Fig. 2:**
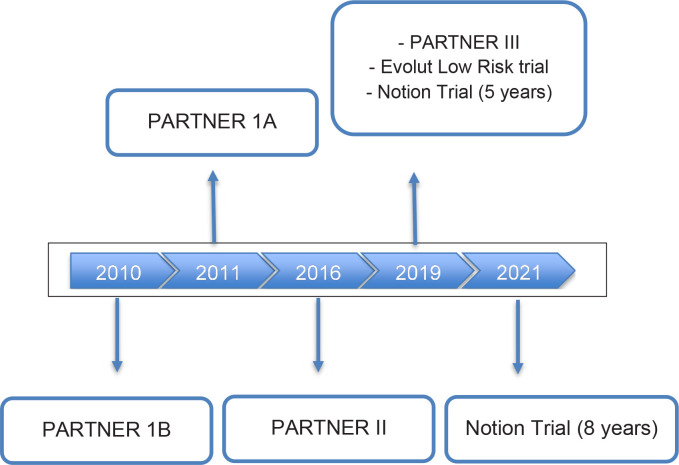
Overview of transcatheter aortic valve replacement trials.

In the Brazilian context, TAVI was recently incorporated into the list of procedures of the Sistema Único de Saúde (the Brazilian unified health system), allowing greater access to the population and demand for the professionals performing TAVI. The expansion of indications, changes in access (transfemoral) as well as literature data point towards the expansion of these techniques, which demands adequate training for surgeons^[6-12]^ (Figure 2).

The endovascular approach to the aorta represents another field of practice for surgeons, with an undeniable Brazilian contribution. In 2020, Gaia et al.^[13]^ published the first case of an Endo-Bentall technique, which consists of the fusion of a customized aortic stent graft positioned in Zone 0 (ascending aorta), coupled to a transcatheter valve in aortic position, with branches to the left and right coronary arteries, with transfemoral and transapical approach, in a patient considered inoperable. Such therapy would not be possible without the understanding that disruptive technology, continuous training, and cooperation are pillars for maintaining the forefront of a specialty.

In this context, Barbosa et al.^[14]^ point out that endovascular surgery should be included in the resident’s training, with the possibility of an additional year in this area. However, which centers provide training in endovascular surgery within the residency program in cardiovascular surgery? And which ones offer fellowships in this area? Such discussion can and should be extended to transcatheter procedures.

## TRAINING

For more than a decade, changes in the medical residency program in cardiovascular surgery have been discussed in Brazil, given the emergence of new technologies and therapies. In 2006, in an editorial to the Brazilian Journal of Cardiovascular Surgery (BJCVS), Prof. Gilberto Barbosa mentioned the “new cardiovascular surgeon”, capable of mastering imaging methods and interventionist skills, in addition to traditional treatment methods, recalling the importance of adapting to the new era^[15]^. The change was consolidated 10 years later, under Prof. Fabio Jatene board^[16]^, with the first group of this new program finishing their training in 2023.

The new program, established as of March 1, 2018, has five years of in-service training, with direct access, without the need for a prerequisite in General Surgery, and with a description of competencies per year in its matrix postgraduate year (PGY)1 - PGY-5 (Table 1). In such manner, we can observe that the acquisition of catheter-based skills is established already in the first year of residency, and at the end of cardiovascular surgery training (PGY-5), the resident is able to coordinate the surgical team as well as to support the residency program as a supervisor.

**Table 1:** Cardiovascular surgery residency program competencies.

PGY-1	PGY-5
To develop the minimum skills necessary for surgical activity	To decide the need to apply scientifically accepted technical variants in order to solve unexpected difficulties
To use the diagnostic methods used in cardiology	To plan and master the execution of the steps of a given procedure in a sequential and organized manner
To use catheters in hemodynamics and to interpret cardiac, coronary, and vascular radiological anatomy	To master the reconstruction of heart valves after analysis during surgery
To understand the basic principles that guide vascular surgery	To master the installation of mechanical circulatory support systems by different routes
To use video technique in cardiovascular and thoracic surgery	To master and perform the different techniques of aortic reconstruction with tubular prostheses or with the use of intraluminal expandable prostheses
To interpret the pathophysiology of cardiopulmonary bypass	To analyze the indications for heart transplantation, the criteria for brain death, and the selection of donors and recipients
To understand and analyze the principles of thoracic surgery	To master the execution of the less complex, palliative, and curative techniques in congenital surgeries
To treat the main cardiac arrhythmias	
To interpret causes of bleeding	To recognize and analyze the most frequent complications of
To dominate the causes of surgical infection	pediatric cardiovascular surgery and ways to solve them
To diagnose and treat cardiogenic shock	

PGY=postgraduate year

It is important to highlight that at the end of the training, the resident can enroll in the Sociedade Brasileira de Cirurgia Cardiovascular (SBCCV) board examination. The applicant must have a minimum number of 100 cardiovascular surgeries with and without the use of cardiopulmonary bypass. However, how many residents finish their training with this minimum number of surgeries? This is a point that must be discussed among the cardiovascular surgery departments in the country.

Wick et al.^[19]^, when evaluating cardiac surgery training in Germany, describe that surgical skills are obtained in the first years of training, helping with procedures. As the resident acquires more responsibilities in the operating room, and under the support and supervision of other surgeons, a minimum number of procedures must be performed, such as coronary artery bypass grafting and valve surgeries, including transcatheter procedures and reoperations.

Juanda et al.^[20]^, in a Canadian study involving 110 residents, found that there was great variability in the contact of residents with interventionist training. Within the assessed sample, approximately 88% reported a need for a larger exposure to rotations that would corroborate the development of skills with catheters. These should be more intensively discussed after the formation of the first groups in our new program as well as the competences established in the new matrix.

Thus, current training in cardiovascular surgery has undergone major changes. The history of the development of aortic stents and the recent expansion of indications for transcatheter valve implants will impact future generations, shaping the training of new cardiovascular surgeons as more challenging. We can and should learn from the past, incorporating new therapeutic options into our daily practice, as protagonists in this process^[21]^.

Surgical procedures are becoming less and less invasive, forcing surgeons to acquire such skills and to perform such procedures. As with percutaneous coronary intervention (PCI), “we should not be fooled again”. With reference to the work carried out by Juanda et al.^[20]^, residents are increasingly demanding exposure to these procedures, which ideally should still happen in their formal training, in a similar way and with the necessary expertise that occurred with vascular surgery^[22]^.

With “the train has left”, begins the paper published by Nguyen et al.^[23]^, when analyzing the treatment of structural heart diseases by cardiovascular surgeons. Some challenges are presented in this discussion: the lack of access to formal training programs in structural diseases, the fact that transcatheter rotations have great variability, even in our national context, with insufficient time to acquire the necessary skills, and resistance in allowing participation in these procedures, particularly due to competition with other specialties. In fact, interventional cardiology programs offer an additional year in structural diseases (in addition to greater exposure in the formal training of these specialists), and the deficit in this standardization is responsible for producing greater discrepancies in terms of experience and exposure of surgeons in training.

In this sense, it is necessary to “think outside the box” and to discuss solutions to correct the delay and deficiency in endovascular and transcatheter training in Brazil, especially in cardiovascular surgery. Kaneko^[24]^ describes cross-training, a period of six to 12 months in interventional cardiology after formal cardiac surgery training. Kilcoyne et al.^[25]^ detail a proposed curriculum for training in structural diseases, ranging from planning, case selection, main procedures (TAVI, MitraClip®, thoracic endovascular aortic repair, PCI), and correlated procedures (transseptal access, insertion of cerebral protection devices, closure of paraprosthetic leaks, and use of alternative accesses such as the transaxillary and transcarotid).

**Fig. 3:**
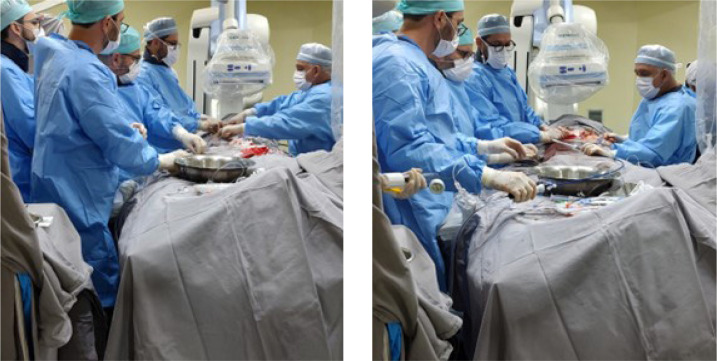
Prof. Dr. José Honório Palma and surgical team of structural heart diseases at the Instituto do Coração of the Hospital das Clínicas of the Faculdade de Medicina of the Universidade de São Paulo during transcatheter aortic valve implantation in the hybrid room.

In the Brazilian context, we have observed some important initiatives that help in the training of our surgeons and residents, among them, the certification in TAVI by SBCCV and the TVI Symposium - Transcatheter Valve Intervention, held in Porto Alegre, which brought together a true Heart Team in structural heart diseases. In addition, centers of excellence such as the Instituto do Coração of the Hospital das Clínicas of the Faculdade de Medicina of the Universidade de São Paulo, under the supervision of Prof. Dr. José Honório Palma, annually offer fellowship opportunities in structural heart diseases in cardiovascular surgery (Figure 3).

Finally, in Brazil, we had a revision of the medical residency program in response to the evolutions that the specialty is facing. However, such a revision still requires adjustments, which leads us to raise some propositions:

- The establishment of a national logbook, with the number of procedures performed by residents in cardiovascular surgery, which would allow evaluating possible deficits in training.
- Expansion of the activities of the Department of Endovascular Surgery of the SBCCV, through courses and training.
- A continuing education program for cardiovascular surgery residents, focused on minimally invasive techniques, endovascular surgery, structural heart disease, and scientific research, in addition to updates in areas such as coronary, valve, and aorta (rotations of two or three months).
- Institutional exchange, allowing residents to observe and participate in procedures not available at their institution.

In addition to these suggestions, the discussion of a national cross-training between cardiovascular surgery and interventional cardiology would be fundamental. In the last year of cardiovascular surgery residency (PGY-5), those who wish to pursue this area could choose to undergo training focused on structural heart diseases in centers that have an adequate volume of procedures.

## CONCLUSION

The impact of transcatheter technologies for surgeons will be significant, and the discussion of measures to improve the training of cardiovascular surgery residents in Brazil is a sine qua non for maintaining the vanguard of the specialty. The challenges are on the table, it is up to all cardiovascular surgeons to understand that adaptation, evolution, and cooperation are the foundations that will sustain Brazilian cardiovascular surgery.

**Table:** 

Author's Roles & Responsibilities	
ASM	Substantial contributions to the design of the work; and the acquisition, analysis, and interpretation of data for the work; drafting the work; final approval of the version to be published
KJSS	Substantial contributions to the design of the work; and the acquisition, analysis, and interpretation of data for the work; drafting the work; final approval of the version to be published
PEUA	Substantial contributions to the design of the work; and the acquisition, analysis, and interpretation of data for the work; drafting the work; final approval of the version to be published
FVAS	Substantial contributions to the design of the work; and the acquisition, analysis, and interpretation of data for the work; drafting the work; final approval of the version to be published
DHM	Substantial contributions to the design of the work; and the acquisition, analysis, and interpretation of data for the work; drafting the work; final approval of the version to be published
MHBC	Substantial contributions to the design of the work; and the acquisition, analysis, and interpretation of data for the work; drafting the work; final approval of the version to be published
JHAPF	Substantial contributions to the design of the work; and the acquisition, analysis, and interpretation of data for the work; drafting the work; final approval of the version to be published
